# Critical Role of an Antiviral Stress Granule Containing RIG-I and PKR in Viral Detection and Innate Immunity

**DOI:** 10.1371/journal.pone.0043031

**Published:** 2012-08-13

**Authors:** Koji Onomoto, Michihiko Jogi, Ji-Seung Yoo, Ryo Narita, Shiho Morimoto, Azumi Takemura, Suryaprakash Sambhara, Atushi Kawaguchi, Suguru Osari, Kyosuke Nagata, Tomoh Matsumiya, Hideo Namiki, Mitsutoshi Yoneyama, Takashi Fujita

**Affiliations:** 1 Laboratory of Molecular Genetics, Institute for Virus Research, Kyoto University, Kyoto, Japan; 2 Research Institute for Science and Engineering, Waseda University, Tokyo, Japan; 3 Laboratory of Molecular Cell Biology, Graduate School of Biostudies, Kyoto University, Kyoto, Japan; 4 Division of Molecular Immunology, Medical Mycology Research Center, Chiba University, Chuo-ku, Chiba, Japan; 5 Influenza Division, Centers for Disease Control and Prevention, Atlanta, Georgia, United States of America; 6 Department of Infection Biology, Faculty of Medicine and Graduate School of Comprehensive Human Sciences, University of Tsukuba, Tsukuba, Japan; 7 Kitasato Institute for Life Sciences, Kitasato University, Tokyo, Japan; 8 Department of Vascular Biology, Institute of Brain Science, Graduate School of Medicine, Hirosaki University, Aomori, Japan; 9 Graduate School of Science and Engineering, Waseda University, Tokyo, Japan; 10 PRESTO, Japan Science and Technology Agency, Honcho Kawaguchi, Saitama, Japan; Keio University, Japan

## Abstract

Retinoic acid inducible gene I (RIG-I)-like receptors (RLRs) function as cytoplasmic sensors for viral RNA to initiate antiviral responses including type I interferon (IFN) production. It has been unclear how RIG-I encounters and senses viral RNA. To address this issue, we examined intracellular localization of RIG-I in response to viral infection using newly generated anti-RIG-I antibody. Immunohistochemical analysis revealed that RLRs localized in virus-induced granules containing stress granule (SG) markers together with viral RNA and antiviral proteins. Because of similarity in morphology and components, we termed these aggregates antiviral stress granules (avSGs). Influenza A virus (IAV) deficient in non-structural protein 1 (NS1) efficiently generated avSGs as well as IFN, however IAV encoding NS1 produced little. Inhibition of avSGs formation by removal of either the SG component or double-stranded RNA (dsRNA)-dependent protein kinase (PKR) resulted in diminished IFN production and concomitant enhancement of viral replication. Furthermore, we observed that transfection of dsRNA resulted in IFN production in an avSGs-dependent manner. These results strongly suggest that the avSG is the locus for non-self RNA sensing and the orchestration of multiple proteins is critical in the triggering of antiviral responses.

## Introduction

Type I and III interferons (IFNs) are cytokines with strong antiviral activity [1], [2]. Upon the binding of IFNs with their cognate receptor complexes, an intracellular signal is activated resulting in the activation of transcription factors, IFN stimulated gene factor 3, heterotrimer of signal transducer and activator of transcription (STAT)1, STAT2, and IFN regulatory factor (IRF)-9, and STAT1 homodimer. These factors induce the activation of hundreds of interferon stimulated genes (ISGs). Some of the ISG products act as antiviral proteins and participate in the blockade of viral replication. The level of double-stranded (ds) RNA-dependent protein kinase (PKR) is enhanced by IFN treatment, however catalytic activity of PKR requires dsRNA. When IFN-treated cells are infected by virus, dsRNA, produced as a by-product of viral replication, activates PKR, and the activated PKR inactivates eukaryotic translation initiation factor (eIF) 2á by phosphorylation [3]. Another antiviral protein 2′–5′ oligoadenylate synthetase (OAS) is also induced to express by IFN. Catalytic activity of OAS requires dsRNA and virus infection activates OAS to produce 2′–5′ A. 2′–5′ A then activates cellular RNase L, and viral RNA is degraded [1]. Although, the “dsRNA-activated inhibition” model is widely accepted, IFN-treated and virus-infected cells do not necessarily undergo suicide, as conventional IFN bioassays have demonstrated IFN-induced survival of infected cells [4]. To explain these phenomena, it has been hypothesized that viral transcription/translation takes place in a specific subcellular compartment, thus the blocking of translation and the degradation of RNA by these antiviral proteins little affect host metabolism. However, no one has yet demonstrated such a compartment.

IFNs are not normally produced at biologically significant levels. Most types of mammalian cells are capable of producing IFN upon viral infection. Viral replication is sensed by cytoplasmic non-self RNA sensors; RIG-I, melanoma differentiation-associated gene 5 (MDA5), and laboratory of genetics and physiology 2 (LGP2), which are collectively termed RLRs, to initiate the cascade of events leading to the activation of transcription factors, IRF-3/-7 and nuclear factor-êB (NF-êB), then the activation of IFN genes [5]–[9]. Thus, the primary function of the IFN system is to sense non-self RNA and to eradicate the invading RNA, which includes RNA derived from the replication of DNA viruses [10], [11]. Although genetic evidence shows that RLR is critical for detecting viral RNA in the cytoplasm, its specific distribution has been unknown.

In this report, we investigated the cellular localization of RIG-I in Influenza A Virus -infected cells. We discovered that viral infection or the transfection of viral RNA causes RIG-I to form granular aggregates containing stress granule markers, which we term antiviral stress granules (avSGs). Our analyses revealed that avSGs are critical for signaling to activate the IFN gene, suggesting that the avSG serves as a platform for the sensing of non-self RNA by RLRs. Furthermore, because the granule also recruits PKR, OAS and RNase L, it is strongly suggested to be the compartment where some antiviral proteins inhibit viral replication.

## Results

### Infection of NS1-deficient IAV Produces Granules Containing RIG-I

**Figure 1:**
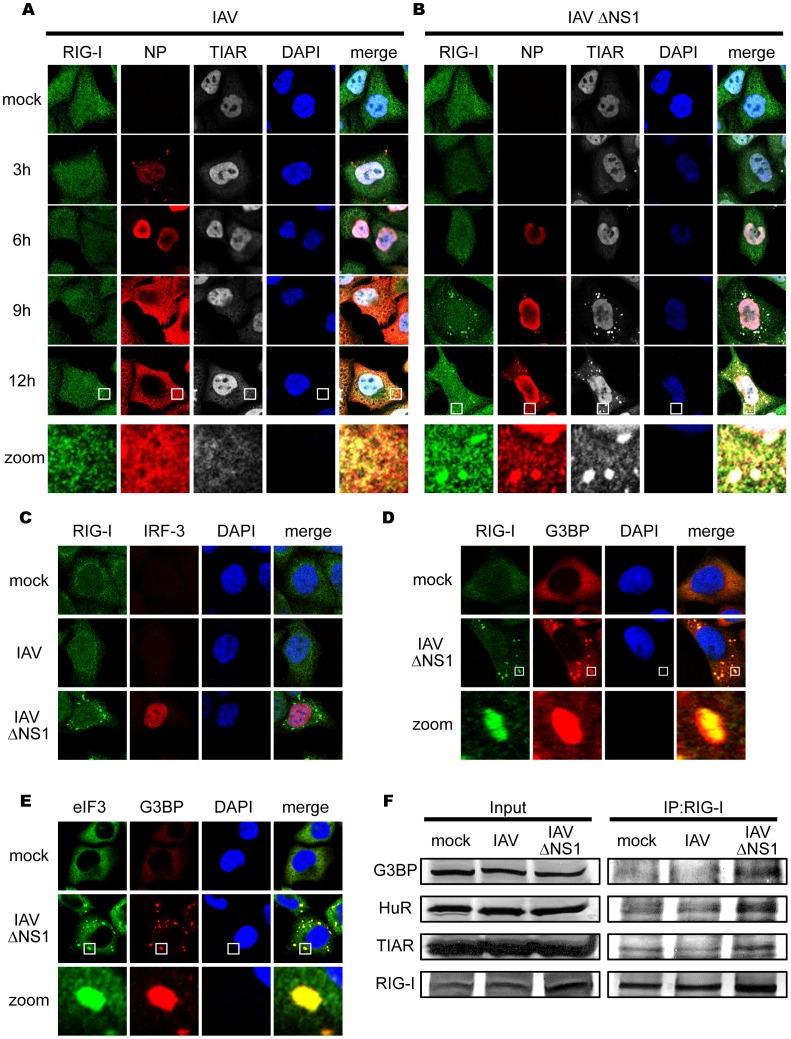
IAV infection causes a speckle-like distribution of RIG-I and stress granule markers. (**A–C**) HeLa cells were mock-treated or infected with IAV (**A**) or IAVÄNS1 (**B**) for the indicated period, fixed, stained, and analyzed with confocal microscopy. The cells were stained with anti-RIG-I (RIG-I), anti-IAV nucleocapsid protein (NP), and anti-TIAR (TIAR) antibodies. Nuclei were stained with DAPI. At 9 h and 12 h after infection, the percentage of speckle-like distribution of RIG-I was 0.5% and 0.0% in IAV-infected cells, and 62.4% and 83.6% in IAVÄNS1-infected cells, respectively. The zoomed images correspond to the boxed region in each panel. The cells at 9 h post infection were stained with anti-RIG-I and anti-IRF-3 antibodies (**C**). (**D and E**) HeLa cells were infected with IAVÄNS1 for 9 h, and stained with anti-G3BP (G3BP), together with anti-RIG-I (RIG-I) (**D**) or anti-eIF3 (eIF3) (**E**). The zoomed images correspond to the boxed regions. (**F**) HeLa cells were mock-treated or infected with IAV or IAVÄNS1 for 12 h. Cell extracts were prepared and immunoprecipitated with anti-RIG-I antibody. The precipitates were analyzed by immunoblotting (IP:RIG-I) using antibody against G3BP, HuR, TIAR and RIG-I. Input: 1/50 of the extracts used for immunoprecipitation were analyzed similarly by immunoblotting.

We generated an anti-RIG-I antibody, which specifically detects RIG-I by immunostaining and immunoblotting (Figure S1) (Materials and Methods) [12]. To observe the cellular distribution of RIG-I, cells were infected with two types of IAV, the wild type (WT) and ÄNS1 which lacks the gene for non-structural protein 1 (NS1), a potent inhibitor of IFN production [13]. WT IAV replication was detectable at 3 h after infection as a nuclear accumulation of viral nucleocapsid protein (NP) (Figure 1A). Later in the infection (9–12 h), NP, presumably as a complex with viral genomic RNA [14], translocated to the cytoplasm. RIG-I was dispersed in uninfected cells and WT IAV infection did not cause any change in its distribution. On the other hand, in cells infected with IAVÄNS1, NP accumulated in the nucleus at 6 h post-infection, however only a fraction of NP translocated to the cytoplasm at 9–12 h (Figure 1B). Unlike WT IAV, the NP of IAVÄNS1 exhibited a speckle-like distribution in the cytoplasm. Notably, formation of this RIG-I-containing speckle strongly correlates with activation of RIG-I-mediated signal activation as judged by nuclear localization (Figure 1C) and dimerization of IRF-3 and concomitant enhanced production of ISGs, such as RLRs and STAT1 (Data not shown). Indeed, the ratio of cells with IAV- and IAVÄNS1-induced nuclear IRF-3 was 2.7% and 33.7%, respectively, and cells containing RIG-I speckles together with nuclear IRF-3 were 0.0% (IAV) and 72.2% (IAVÄNS1).

### IAVÄNS1-induced Granules Contain Both Stress Granule Marker and Anti-viral Proteins

We characterized the nature of these speckles by using various antibodies and found that interestingly, RIG-I exhibited co-localization with NP (83.5%) and a stress granule (SG) marker, T-cell restricted intracellular antigen-related protein (TIAR) (97.1%) at 9 h (Figure 1B). Other SG markers are similarly recruited to the granules produced by IAVÄNS1: Ras-GAP SH3 domain-binding protein (G3BP) (97.6% colocalized with RIG-I), eIF3 (99.8% colocalized with G3BP) (Figure 1D and 1E), and human antigen R (HuR) (98.8% colocalized with RIG-I) (data not shown). Furthermore, physical interaction between RIG-I and SG markers was demonstrated by pull-down assays (Figure 1F). These results strongly suggest that although IAV infection potentially induces signaling to activate the IFN gene and the formation of granular aggregates containing SG markers, NS1 strongly blocks both. Ectopic expression of full-length NS1 and the N-terminal RNA-binding domain of NS1 dramatically inhibited both granule-formation and RIG-I signaling in response to IAVÄNS1 infection, indicating that the N-terminal domain of NS1 is responsible for these activities (Figure S2A and S2B).

**Figure 2:**
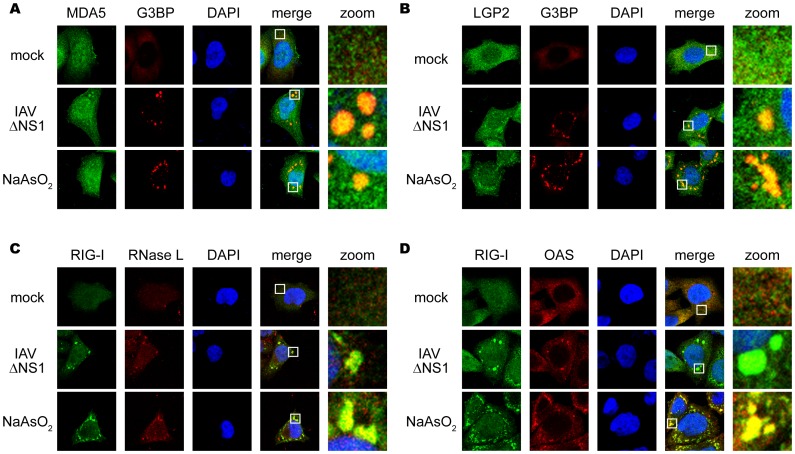
Antiviral proteins are colocalized with SGs. (**A–D**) HeLa cells were mock-treated (mock), infected with IAVÄNS1 for 9 h, or treated with NaAsO_2_ for 1 h. Cells were fixed and stained for G3BP and MDA5 (94.2% colocalization) (**A**), G3BP and LGP2 (97.6% colocalization) (**B**), RIG-I and RNase L (84.5% colocalization) (**C**), RIG-I and OAS (87.4% colocalization) (**D**) in IAVÄNS1-infected cells. The zoomed images correspond to the boxed regions.

SGs are an intracellular ribonucleoprotein (RNP) complex generated by cellular stress, including oxidative, heat shock, and endoplasmic reticulum stress, and contain translation-stalled mRNAs and various RNA-binding proteins [15]. Because many of the SG markers are RNA-binding proteins, we examined the localization of other RLRs, MDA5, and LGP2, as well as PKR (see below), RNase L, and OAS by using specific antibodies. Interestingly, these proteins were also recruited to SG and virus-induced granules in response to arsenite and IAVÄNS1, respectively (Figure 2A, 2B, 2C, 2D).

### IAVÄNS1 Infection Induces Antiviral SGs Containing Viral RNA

**Figure 3:**
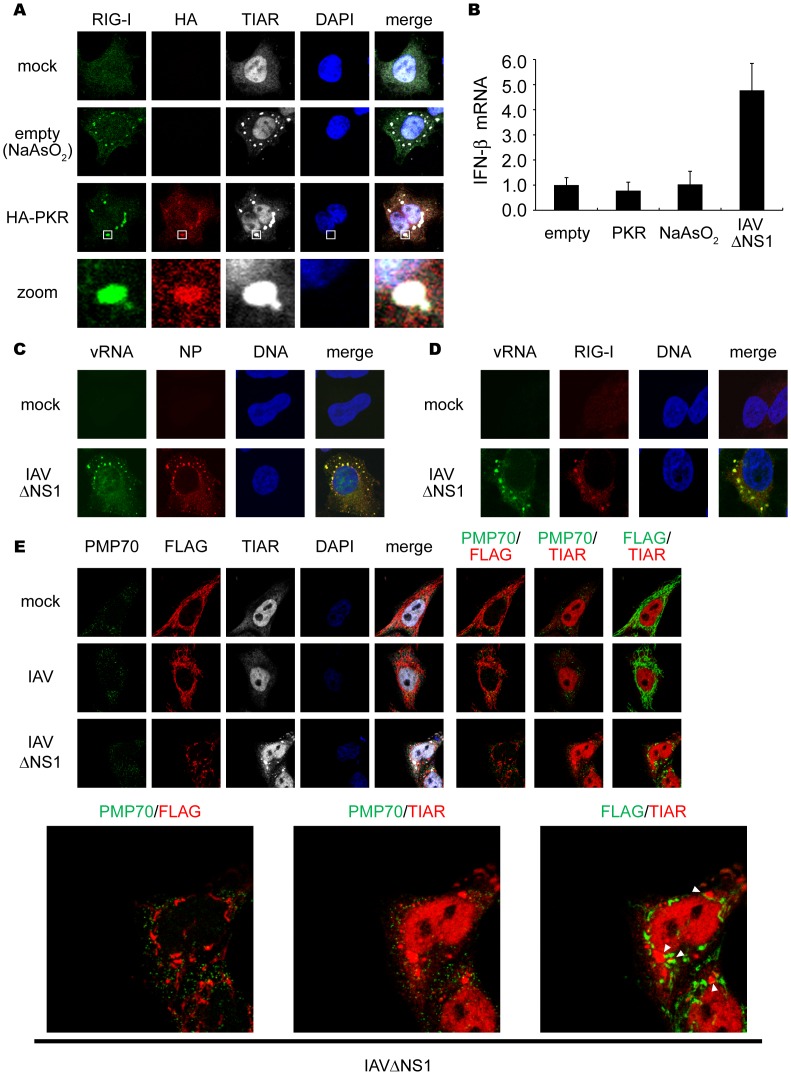
Viral RNA is required for formation of functional SG to activate RIG-I/IPS-1 signaling pathway. (**A**) 293T cells were transfected with empty vector (empty) or the HA-PKR expression vector (HA-PKR) for 24 h, or treated with NaAsO_2_ for 1 h and stained with anti-RIG-I, anti-HA (PKR) and anti-TIAR antibodies and DAPI. The zoomed images correspond to the boxed regions. (**B**) 293T cells were transfected with empty vector (empty), or the HA-PKR expression vector for 48 h, or treated with NaAsO_2_ for 1 h, or infected with IAVÄNS1 for 12 h. Relative mRNA levels of endogenous IFN-â gene were determined by quantitative PCR (qPCR). Data are represented as the mean standard ± error of the mean (SEM). (**C and D**) HeLa cells were mock-treated or infected with IAVÄNS1 for 12 h. Viral RNA (vRNA) was detected by the FISH method using an RNA probe complementary to the segment 1 of the IAV, and NP (**C**) and RIG-I (**D**) were detected using anti-NP and anti-RIG-I antibodies (97.1%, and 98.2% colocalization of vRNA with NP and RIG-I, respectively). TO-PRO-3 was used for staining of nuclear DNA (DNA). (**E**) HeLa cell lines stably expressing FLAG-tagged IPS-1 were mock-treated or infected with IAV or IAVÄNS1 for 10 h. The cells were stained with anti-PMP70, anti-FLAG, and anti-TIAR antibodies. The white arrowheads indicated the contacts between FLAG-IPS-1 and TIAR. 67.8% of IAVÄNS1 infected cells exhibited contacts, whereas IAV infected cells hardly exhibited the contact (2.7%). The zoomed images of PMP70 (Green) and FLAG (Red), PMP70 (Green) and TIAR (Red), and FLAG (Green) and TIAR (Red) in IAVÄNS1-infected cells were shown in the bottom panel.

These results prompted us to examine correlation between the formation of SGs and activation of the IFN gene. Treatment of cells with arsenite (NaAsO_2_), which induced oxidative stress, produced granules similar to those generated by IAVÄNS1 (Figure 3A). Similarly, artificial overexpression of PKR resulted in the formation of SGs (58.9%) (Figure 3A). Although IAVÄNS1 infection activated the IFN-â gene, neither arsenite nor PKR activated the gene (Figure 3B). These results suggest that SGs and virus-induced granules are functionally distinct, possibly due to the presence of viral RNA in virus-induced granules. Indeed, fluorescence *in situ* hybridization (FISH) clearly demonstrated that viral RNA colocalized with the granules of NP (Figure 3C) and RIG-I (Figure 3D) in IAVÄNS1-infected cells whereas IAV-infected cells showed colocalization of viral RNA with NP (Figure S3A) but not with RIG-I (Figure S3B). Once viral RNA is engaged by RIG-I-containing complex, IFN-â promoter stimulator-1 (IPS-1, also known as MAVS, VISA or Cardif) expressed on the outer membrane of mitochondria is recruited to facilitate RIG-I-IPS1 signaling in a Mitofusin 1-dependent manner [12], [16]. Although most of IPS-1 localizes on mitochondrial network in uninfected and IAV-infected cells, IAVÄNS1 infection induces speckle-like distribution. The re-localized IPS-1 exhibits apparent contacts with TIAR- (Figure 3E, bottom-right panel), and RIG-I- (Figure S4) containing SGs. This is consistent with a model that SG physically contacts with mitochondrion mediated by interaction between RIG-I and IPS-1 through caspase recruitment domain (CARD)-CARD homotypic interaction [17]–[20]. Although association of FLAG-tagged IPS-1 with peroxisome membrane protein (PMP70)-positive peroxisomes after viral infection was reported [21], we did not observe their apparent association (Figure 3E).

SG production is not a result of RIG-I signaling or IFN signaling because overexpression of IPS-1 activated the nuclear translocation of IRF-3 without generating SGs (Figure S5A), and SGs formed in IFN receptor-deficient HEC-1B cells (Figure S5B). Furthermore, other viruses including Sindbis (SINV), encephalomyocarditis (EMCV), and Adeno (Ad) dl203 viruses also generated granules containing RIG-I and G3BP (Figure S5C), suggesting this SG-like granule to be a general response to viral infections. To distinguish virus-induced granules from conventional SGs, we termed the virus-induced speckles as antiviral SGs (avSGs).

### Impairment of Formation of avSGs Inhibits IAVÄNS1-induced IFN Activation

**Figure 4:**
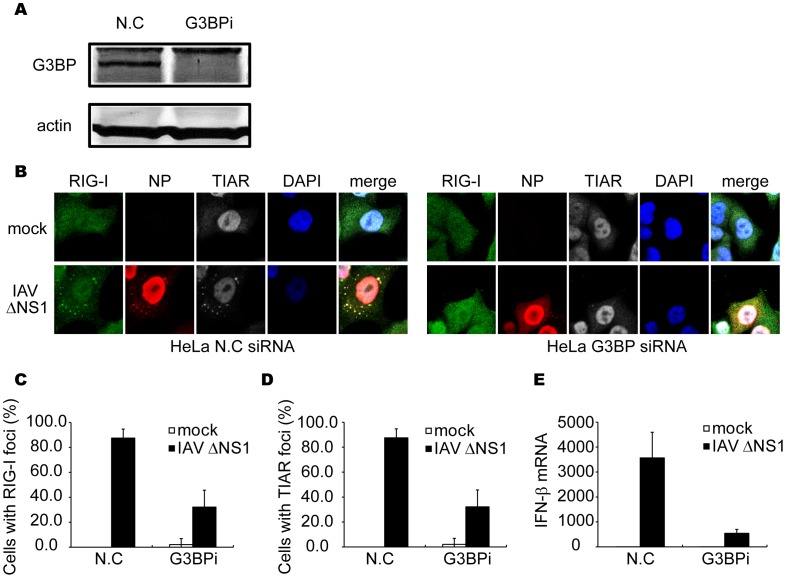
Knockdown of G3BP impairs formation of avSG and IFN-â gene activation. (**A–E**) HeLa cells were transfected with control siRNA (N.C) or siRNA targeting human G3BP (G3BPi). At 48 h after transfection, cells were harvested and G3BP and actin were detected by immunoblotting (**A**). Cells were mock-treated (mock) or infected with IAVÄNS1 for 12 h and fixed and stained with anti-RIG-I, anti-NP and anti-TIAR antibodies and DAPI (**B**). The percentage of cells containing foci of RIG-I (**C**) or TIAR (**D**) was determined. Relative mRNA level of IFN-â was determined by qPCR (**E**). Data are represented as the mean standard ± error of the mean (SEM).

In order to address whether the formation of avSGs is required for IFN expression, we knocked down G3BP, a critical component for formation of the canonical SGs. G3BP siRNA clearly down-regulated G3BP expression (Figure 4A). Consistent with a previous study [22], the knockdown of G3BP strongly inhibited avSG formation in IAVÄNS1-infected cells (Figure 4B), and the number of cells which showed a speckle-like distribution of RIG-I and TIAR was diminished (Figure 4C and 4D). Moreover, IFN-â gene expression was strongly inhibited in G3BP knockdown cells compared to control siRNA-treated cells (Figure 4E). These results suggest that avSG formation is required for efficient activation of type I IFN. Furthermore, knockdown of eIF3 or RHAU, both of which are components of SG, also blocked both avSG formation and IFN gene activation (data not shown).

### IAVÄNS1 Infection Induces PKR’s Activation and Accumulation in avSGs

**Figure 5:**
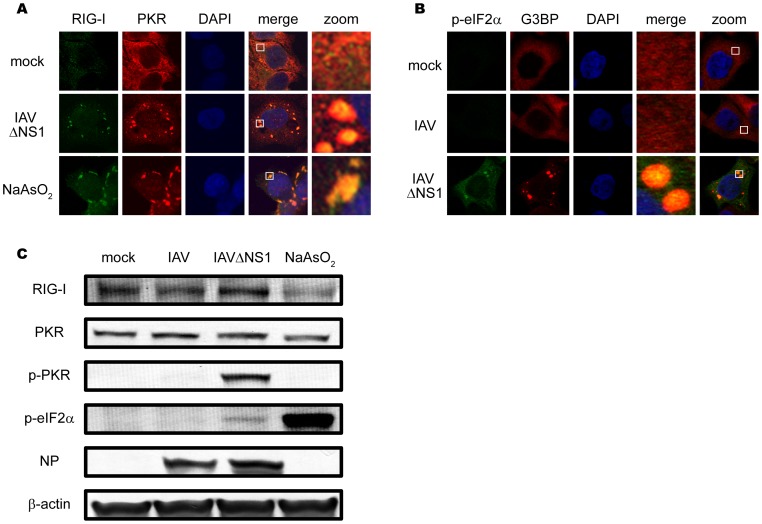
Localization and activation of PKR in IAVÄNS1-induced avSGs. (**A**) HeLa cells were mock-treated or infected with IAVÄNS1 for 9 h or treated with NaAsO_2_ for 1 h. Cells were fixed and stained with anti-RIG-I and anti-PKR antibodies (% of colocalization: 95.1% and 97.0% in IAVÄNS1-infected and NaAsO_2_-treated cells, respectively). The zoomed images correspond to the boxed regions. (**B**) HeLa cells were mock-treated or infected with IAV or IAVÄNS1 for 9 h and stained with anti-phospho-eIF2á (Ser 51) (p-eIF2á) and G3BP (% colocalization: 0.0% and 46.5% in IAV and IAVÄNS1-infected cells, respectively). The zoomed images correspond to the boxed regions. (**C**) HeLa cells were infected with IAV or IAVÄNS1 for 12 h or treated with NaAsO_2_ for 1 h. Cell extracts were prepared and subjected to SDS-PAGE, and immunoblotted using antibodies against RIG-I, PKR, phosphorylated PKR (Thr 446) (p-PKR), phosphorylated eIF2á (Ser 51) (p-eIF2á), IAV NP, and â-actin.

It has been proposed that a family of protein kinases including PKR, general control non-derepressible 2 (GCN2), PKR-like endoplasmic reticulum kinase (PERK), and heme-regulated eIF2á kinase (HRI) phosphorylate eIF2á, resulting in formation of SGs. Arsenite treatment causes oxidative stress leading to the activation of HRI, and SGs are produced. PKR is activated by dsRNA or 5¢ppp-containing RNA [23], therefore we speculate that the IAV RNA activates PKR resulting in the formation of avSGs via the phosphorylation of eIF2á. To address the involvement of PKR, we examined the localization of PKR in arsenite-treated and IAVÄNS1-infected cells and found that PKR accumulated in SGs and avSGs (Figure 5A). Interestingly, phosphorylated eIF2á was also detected in avSGs specifically generated by IAVÄNS1 but not in IAV-infected cells (Figure 5B). Immunoblotting confirmed that IAVÄNS1 specifically induced the phosphorylation of PKR and eIF2á whereas arsenite treatment induced the phosphorylation of eIF2á without PKR activation, indicating that IAVÄNS1 and arsenite induce SGs via distinct pathways (Figure 5C).

### Critical Role of PKR in avSG Formation and IFN Production in IAV-infected Cells

**Figure 6:**
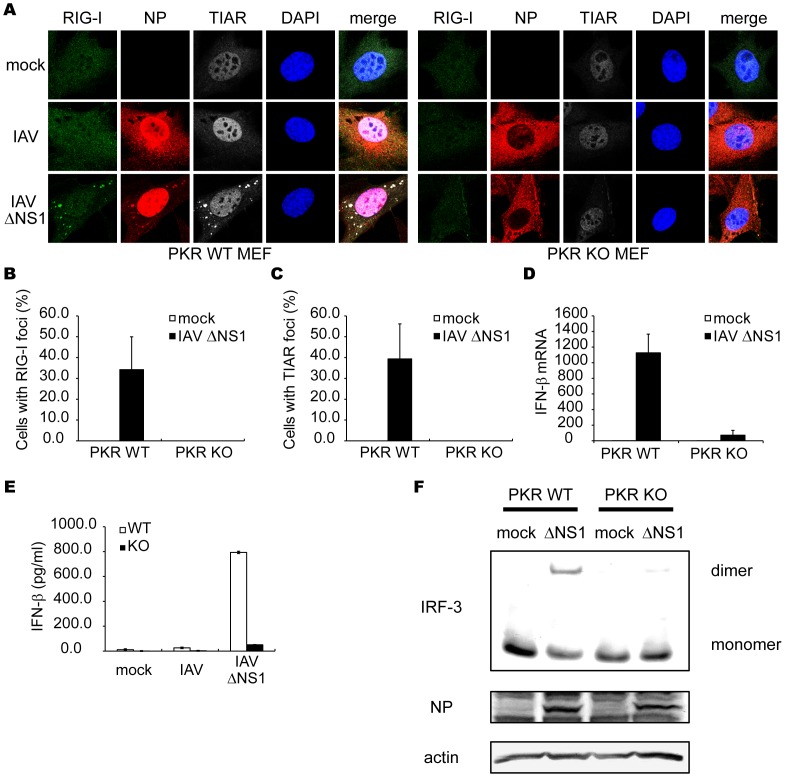
Critical role of PKR in formation of avSG and IFN-â gene activation. (**A–C**) MEFs derived from WT and PKR KO mice were mock-treated or infected with IAVÄNS1 for 12 h. The cells were stained with anti-RIG-I, anti-IAV NP and anti-TIAR antibodies and DAPI (**A**). The percentage of cells containing foci of RIG-I (**B**) or TIAR (**C**) was determined. (**D–F**) PKR WT and PKR KO MEFs were mock-treated or infected with IAVÄNS1. The IFN-â mRNA level at 9 h post-infection was determined by qPCR (**D**). The IFN-â protein levels in culture medium at 15 h post-infection were quantified by ELISA (**E**)**.** Cell extracts were subjected to Native-PAGE and IRF-3 dimer was detected by immunoblotting using anti-IRF-3 antibody. IAV NP and actin were detected by SDS-PAGE followed by blotting using anti-NP and anti-actin antibodies (**F**). Data shown in B-E are represented as the mean standard ± error of the mean (SEM).

**Figure 7:**
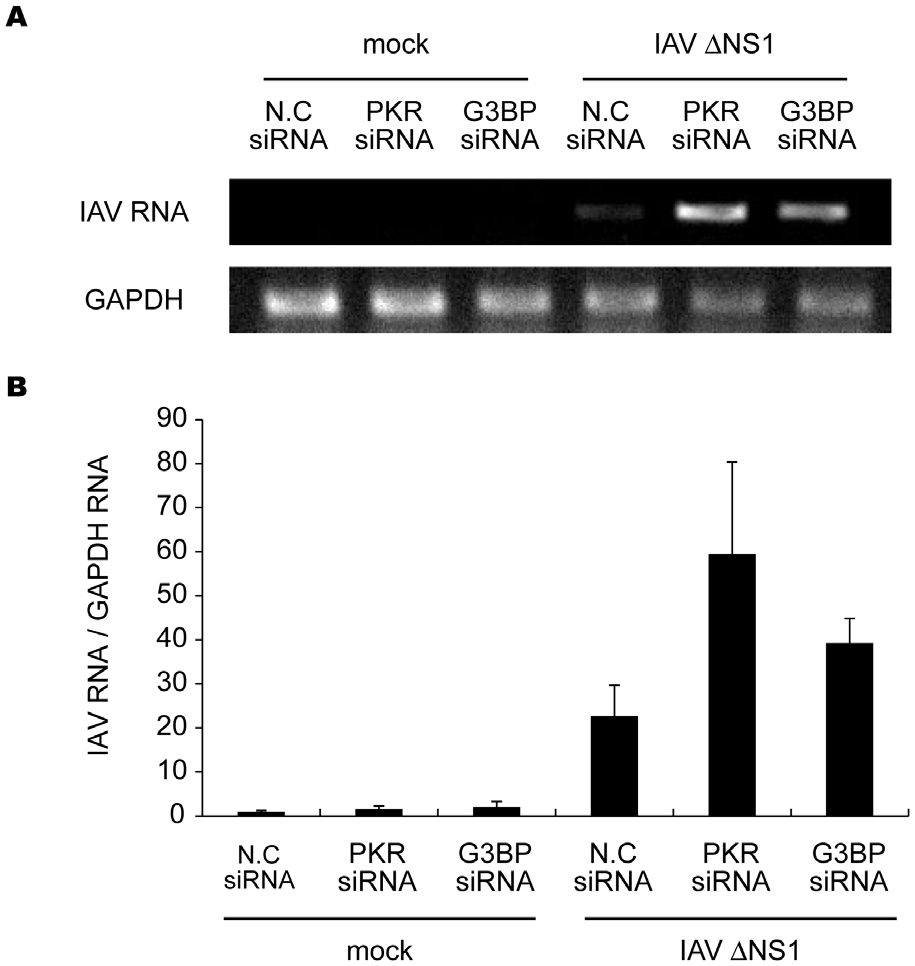
Inhibition of avSG formation enhanced IAV viral replication. HeLa cells were transfected with control siRNA (N.C) or siRNA targeting human PKR mRNA or G3BP. At 48 h after transfection, cells were mock-treated or infected with IAVÄNS1 for 24 h. The expression levels of IAV RNA segment 3 and GAPDH mRNA were determined by RT-PCR (**top**). The IAV RNA expression patterns were quantified with LAS-1000 UV mini (Fujifilm, Japan) and normalized with GAPDH (**bottom**). Data are represented as the mean standard ± error of the mean (SEM).

The results described above indicate the formation of viral RNA-containing avSGs to be essential to RLR-mediated antiviral signaling. To evaluate the requirement of PKR during IAVÄNS1 infection, we analyzed the formation of avSGs in mouse embryonic fibroblasts (MEFs) derived from WT and PKR knock-out (KO) mice (Figure 6). PKR WT and KO MEFs were infected with either IAV or IAVÄNS1, and stained with the anti-RIG-I, anti-NP, and anti-TIAR antibodies and calculated the frequency of avSGs. In the case of WT IAV, avSGs did not form in WT and KO MEFs as determined in Figure 1. IAVÄNS1 produced avSGs in WT but not in PKR KO MEFs (Figure 6A, 6B, 6C). Moreover, deletion of PKR resulted in a blockade of IFN-â gene expression (Figure 6D), production of IFN-â protein (Figure 6E), and IRF-3 dimerization (Figure 6F). Furthermore, we confirmed these results using siRNA targeting PKR expression in HeLa cells. The siRNA efficiently knocked down endogenous PKR expression, resulting in a strong inhibition of the IAVÄNS1-induced IFN-â gene expression, concomitant impairment of the number of avSG (Figure S6). Taken together, the results indicate that PKR is essential for avSGs and IFN gene activation in IAVÄNS1-infected cells. Importantly, blocking avSG formation by knockdown of PKR or G3BP enhanced the replication of IAVÄNS1 (Figure 7). These results strongly indicate a novel role for avSGs in the antiviral innate immune responses.

It is worth to note that in the absence of PKR, cytoplasmic transport of NP is accelerated (Figure 6A). This effect is also observed in HeLa cells in which PKR is knocked down (Figure S6C). Although the mechanism is unknown, these results suggest that PKR negatively regulate cytoplasmic transport of IAV nucleocapsid.

### Viral RNA Generates avSGs in a PKR-dependent Manner

**Figure 8:**
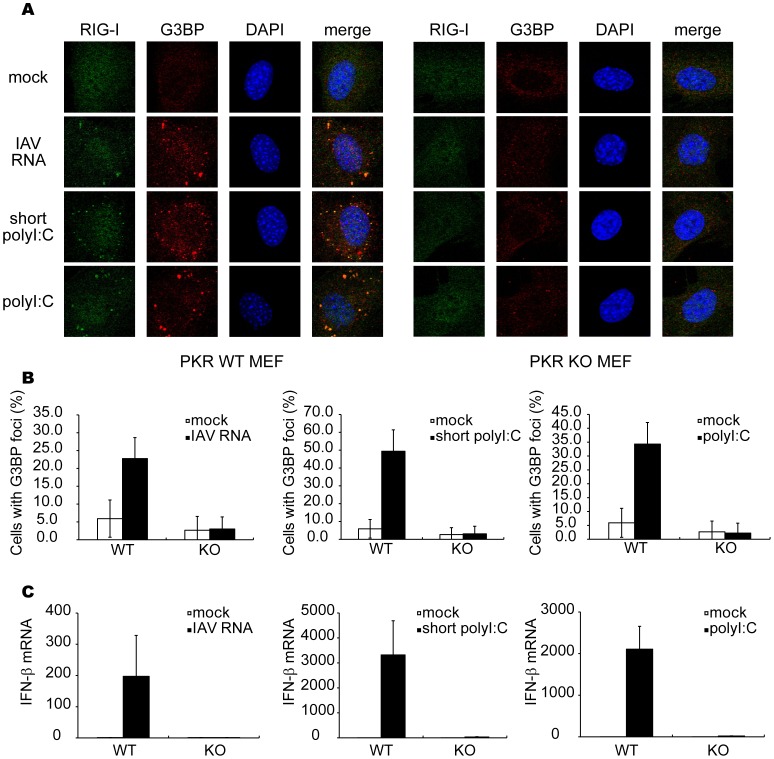
Viral RNA and polyI:C induce formation of avSGs and IFN-â gene expression in a PKR-dependent manner. (**A–C**) MEFs derived from WT and PKR KO mice were mock-treated or transfected with IAV genomic RNA, short poly I:C or long poly I:C for 9 h and stained with anti-RIG-I, anti-G3BP antibodies and DAPI (**A**). The percentage of cells containing foci of G3BP was shown in (**B**). Relative IFN-â mRNA levels were determined by qPCR (**C**). Data shown in B and C are represented as the mean standard ± error of the mean (SEM).

Previous reports showed that genomic RNA of IAV is responsible for triggering antiviral signaling via RIG-I [6], [9], [24], [25]. Because the IAV genome is not infectious, we extracted it from the IAV-infected cells and transfected it into WT or PKR KO MEFs, and investigated whether the IAV genomic RNA solely induces the formation of avSGs and subsequent activation of the IFN gene. As shown in Figure 8A, the IAV genomic RNA is sufficient to produce avSGs, indicating that neither viral protein nor viral RNA replication is required. Furthermore, PKR is required for the formation of viral RNA-induced avSGs (Figure 8A and 8B). Because PKR is also required for poly I:C-induced IFN gene activation [26], we tested short and long poly I:C, which selectively activate RIG-I and MDA5, respectively [27]. Short and long poly I:C induced the formation of avSGs in a PKR-dependent manner (Figure 8A and 8B). We confirmed that IFN-â production by these RNA is PKR-dependent (Figure 8C). Viral but not host RNA is capable of triggering the response, as demonstrated by the finding that total RNA extracted from infected cells but not uninfected cells induced the formation of avSGs and activation of the IFN-â gene (Figure S7A and S7B). These findings demonstrate that PKR is necessary for formation of avSGs which recruits viral RNA and RLRs to trigger IFN gene activation during the IAV-infection. Of note, as shown in Figure 3B, overexpression of PKR can activate SG formation but not IFN expression in the absence of viral RNA, suggesting that function of PKR is prerequisite but insufficient for efficient induction of RLR-mediated antiviral signaling.

## Discussion

Recent studies have identified the domain structure of RLRs and the various adaptor proteins regulating RLR-mediated antiviral signaling cascades [28], but how RLRs encounter viral RNA in infected cells remain unclear. In this study, we found that all RLRs are recruited into cytoplasmic granules, termed avSGs, upon viral infections. avSGs contain many SG markers, G3BP, TIAR, and eIF3, but unlike canonical SGs, also contained viral RNA and viral NP. We demonstrated that avSGs are critical to virus-induced IFN gene activation. Since RLRs must efficiently find their ligands to act as vital sensors for viral RNA, avSGs may facilitate a proper encounter between viral RNA and RLRs. In addition, OAS and RNase L are recruited to avSGs, supporting the model that RNase L amplifies IFN-inducing signaling by unearthing cryptic ligands for RIG-I and MDA5 [29]. Furthermore, the specific recruitment of antiviral proteins in avSGs suggests a critical role in the blocking of viral replication without an effect on host normal translation. We also observed that other viruses including SINV, EMCV Adenovirus, Hepatitis C virus and Newcastle disease virus induced avSG (Figure S5C and data not shown), suggesting that avSGs may function as a general platform for detection of many viruses to initiate antiviral signaling. Because PKR alone cannot trigger IFN gene activation (Figure 3B), PKR contributes at upstream of IAV-induced RIG-I activation. It was reported that SINV activates GCN2 to phosphorylate eIF2á [30], suggesting that the several viruses may activate different eIF2á kinases to form avSG.

**Figure 9:**
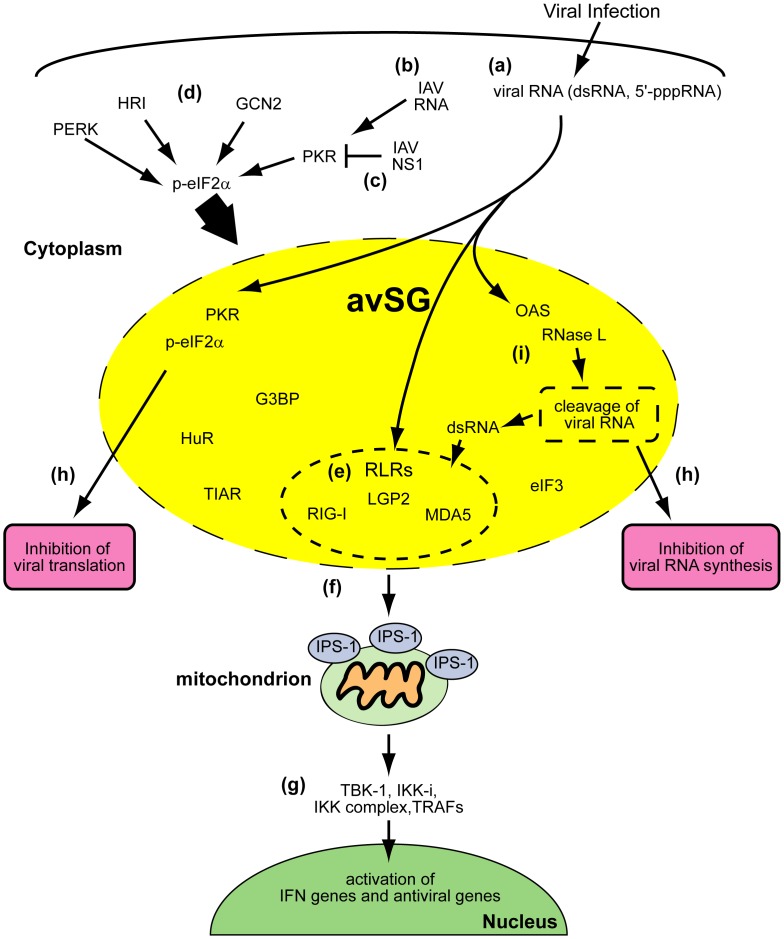
avSGs and innate antiviral responses. Viral infections generate RNA with non-self-signatures such as a 5′-tri-phosphate or double-stranded structure (**a**). In the case of IAVÄNS1, PKR is critical for the formation of avSGs (**b**). Wild-type IAV inhibits formation of avSGs by the actions of the NS1 protein **(c**). Some other viruses may activate different eIF2á kinases, such as GCN2, PERK, and HRI, to produce functional avSGs (**d**). avSGs are composed of SG markers and other RNA-binding proteins including RLRs, antiviral proteins (PKR, OAS, and RNase L) and viral RNA. Within avSGs, viral RNA could be sensed by RLRs to trigger antiviral signaling (**e**). Activated RLRs recruit mitochondrial IPS-1 via CARD-CARD interactions (**f**). IPS-1 serves as another platform for TRAFs and protein kinases, TBK-1, IKKi and IKK complex, to activate target genes (**g**). The antiviral proteins are activated by viral RNA to block viral RNA synthesis and translation (**h**). Moreover, OAS-RNase L system may produce dsRNA to amplify the RLR signaling (**i**).

Our model for the function of avSGs is summarized in Figure 9. We do not strictly rule out a possibility that PKR may directly phosphorylate target molecules to participate IFN gene activation, however because activation of PKR alone is not sufficient to trigger IFN production, this effect may be incremental.

Recognition of viral RNA by RIG-I and MDA5 induces ATP-dependent conformational change of the molecules and allows them to interact with mitochondrial IPS-1 via CARD-CARD interaction. Several reports indicated that activated RIG-I forms dimer or oligomer, which is required for efficient signal activation [31], [32]. Furthermore, it was demonstrated that IPS-1 is redistributed on mitochondria in response to viral infection and IPS-1 forms prion-like aggregates [12], [16]. These observations suggest a possibility that local enrichment of both RLRs and IPS-1 is required for signaling. Although our attempt to detect biochemical interaction between RLR-containing avSG and IPS-1 aggregates *in vitro* has been unsuccessful so far because of insoluble property of them, our immunohistochemical analysis strongly indicates that IPS-1-enriched mitochondria are physically attached with avSG in response to IAVÄNS1 infection (Figure 3E and S4), suggesting critical role of avSG as a platform for RLR-IPS-1 interaction.

The phosphorylation of eIF2á at Ser51 is known to trigger the formation of canonical SGs, however the precise mechanism by which the phosphorylated eIF2á recruits other SG components is not well understood because of difficulty of biochemical analysis [33]. We demonstrated that PKR was activated, recruited to avSGs, and essential for avSGs to form after IAVÄNS1 infection. Viral RNA is primarily responsible for triggering the PKR activation because transfection of IAV genomic RNA or poly I:C induced avSG formation and IFN gene activation in a PKR-dependent manner. Consistent with our data, some studies revealed that PKR deficiency impair production of IFN in response to polyI:C and viral infections [34]–[38]. Schulz et al. reported that PKR is not required for production of IFN-á/â proteins in response to IAV in bone marrow-derived dendritic cells (BM-DCs) [39]. This is apparently inconsistent with our data obtained with MEFs. Although we were unable to directly compare between MEF and BM-DC, our explanation for this discrepancy is cell type difference, because IFN protein expression as determined by ELISA (Figure 6E) is consistent with mRNA level (Figure 6D) and PKR knockdown with HeLa cells dramatically diminished IFN production (Figure S6B). BM-DC may utilize eIF2á kinase other than PKR to form avSG.

The NS1 of IAV is a multifunctional protein that inhibits various host factors, including PKR [40], [41], RIG-I [42], [43], and tripartite motif-containing protein 25 (TRIM25), known to regulate RIG-I activation [44], and potentially sequesters viral dsRNA through its dsRNA-binding domain. Here, we demonstrated that NS1 of IAV markedly inhibited avSGs and the IFN gene’s activation. Consistently, a recent report demonstrated that formation of IAV-induced SG was inhibited by wild type NS1, but not by mutant NS1, in which Arg38 and Arg41 are substituted to Ala [41]. Several classes of viruses are known to inhibit the formation of SGs during infection. The West Nile and Sendai viruses encode RNA that interacts with TIAR and inhibits SG assembly [45], [46]. The poliovirus 3C protease cleaves G3BP at Gln 326 during the infection process [22]. Moreover, Simpon-Holley et al. recently demonstrated that the E3L protein of Vaccinia virus preventing PKR activation and phosphorylation eIF2á and the Vaccinia virus ÄE3L, lacking E3L genes, generated a granular-like structure distinguished from a granule termed antiviral granule (AVG) [47]. They also reported that MEFs lacking the AVG component TIA-1 exhibited increased Vaccinia viral replication, suggesting that AVG is critical for antiviral host responses. These results strongly suggest that viruses acquire means to inhibit the formation of avSGs and subsequent activation of the IFN gene.

The canonical SG has been proposed as a storage compartment for translation-stalled host mRNA in response to various stresses and possible partner with another RNP complex, processing body, which is responsible for mRNA degradation [33]. Therefore, SG has been considered as a compartment for dynamic translational regulation upon environmental stress. Our findings discover a new role for newly identified avSG as a platform for interaction between viral RNA and host antiviral molecules to trigger a cascade of events leading to eradication of the virus. Although the difference between SG and avSG is not fully understood at this point, future research will delineate mechanism of their assembly and biological functions in stresses and immune responses.

## Materials and Methods

### Cell Culture, Transfection, and Viruses

MEFs from *pkr* +/+ and −/− mice were obtained from Dr. Yi-Li Yang [26]. HeLa, 293T, and HEC-1B [48], [49] cells were maintained in Dulbecco’s modified Eagle’s medium (DMEM) with FBS and penicillin-streptomycin (100 U/ml and 100 µg/ml, respectively). HeLa cell line stably expressing FLAG-tagged IPS-1 was described previously [12]. 293T cells were transfected with FuGENE6 (Roche) or Lipofectamine 2000 (Invitrogen). Adenovirus type 12 (Ad12) dl203, provided by Dr. K. Shiroki, and SINV were propagated in 293T cells and Vero cells, respectively. IAV (A/PR/8/34 and ÄNS1), originally produced by Dr. A. Garcia-Sastre (Mount Sinai School of Medicine, USA) and provided by Dr. S. Akira (Osaka University, Japan), were grown in the allantoic cavities of 9-day-old embryonated eggs. Cells were treated with the culture medium (‘mock-treated’) or infected with IAV, IAVÄNS1, SINV, EMCV, or Ad12 dl203 in serum-free and antibiotic-free medium. After adsorption for 1 h at 37°C, the medium was changed and infection was continued for various periods in the presence of serum-containing DMEM. IAV genomic RNA was extracted from partially purified virus stock by TRIzol (Invitrogen) and 1.0 µg of viral RNA was transfected with Lipofectamine RNAi MAX (Invitrogen) in a 35 mm dish. PolyI:C was purchased from Amersham. Short polyI:C was prepared as described previously [27].

### Immunoblotting, Antibodies and Reagents

The preparation of cell extracts and immunoblotting have already been described previously [5], [50]. The polyclonal antibody used to detect human IRF-3 in native PAGE and anti-human and anti-mouse IRF-3 polyclonal antibodies for immunostaining were described previously [50]. The monoclonal antibody against Influenza NP (mAb61A5), which was generated by Dr. Y. Kikuchi (Iwaki Meisei University, Japan), was provided by Dr. F. Momose (Kitasato University, Japan) [51]. The monoclonal antibody against human OAS (6-1) was provided by Dr. Y. Sokawa (Kyoto Institute of Technology, Japan). The anti-human RIG-I, anti-human MDA5, and anti-human LGP2 antibodies were originally generated by immunizing rabbits with a synthetic peptide corresponding to amino acids 793–807 of human RIG-I, 145–160 of human MDA5, and 535–553 of human LGP2, respectively. As shown in Figure S1, knockdown of endogenous RIG-I by shRNAs specifically inhibit granule-like accumulation of RIG-I in immunostaining (Figure S1A) and appearance of band corresponding endogenous RIG-I in western blotting (Figure S1B), indicating high specificity of the anti-RIG-I antibody. Furthermore, a similar staining pattern was obtained with a monoclonal antibody for human RIG-I produced by Perseus Proteomics Inc, Japan. Other antibodies were obtained from the following sources: anti-G3BP (611126) from Transduction Laboratories™, anti-IRF-3 (CBX00167) from COSMO BIO, anti-PKR (sc-6282), anti-RNase L (sc-22870), anti-G3BP (sc-70283), anti-eIF3 (sc-16377), anti-c-myc (sc-40) and anti-TIAR (sc-1749) from Santa Cruz Biotechnology, anti-FLAG (M2) from Sigma, anti-phospho-PKR (pT446) (1120-1) from Epitomics, anti-actin (MAB1501R) from CHEMICON International, anti-PMP70 (ab3421) from Abcam, anti-HA-Tag (6E2) and anti-phospho-eIF2á (Ser51) (119A11) from Cell Signaling, and anti-HuR (RN004P, RIP-Certified antibody) and anti-â-actin (PM053) from MBL. Alexa 488-, 594-, and 633- conjugated anti-mouse, anti-rabbit, or anti-goat IgG antibodies purchased from Invitrogen were used as secondary antibodies. 0.5 mM Sodium arsenite (Sigma) was added to the cell culture for 1 h.

### Plasmid Constructs

The p-125 Luc, pEF-BOS-FLAG-IPS-1, pCAGGS-myc, pCAGGS-myc-NS1, pCAGGS-myc-NS1 (amino acids 1–80), pCAGGS-myc-NS1 (amino acids 81–230) plasmids have been described previously [42], [48], [52]. cDNA for human PKR was obtained from Dr. A. Hovanessian (University Paris, France) and pEF-BOS-HA-PKR and pEF-BOS-HA-IPS1 were obtained by subcloning the cDNA into the vector pEF-BOS, respectively. pSUPER, pCMVÄR8.91, pMDG, and pRDI292 were provided by Dr. D. Trono (Ecole Polytechnique Federale de Lausanne, Switzerland) [53]–[56].

### Luciferase Reporter Assay

The Dual-Luciferase Reporter Assay System (Promega) was used according to the manufacturer’s instructions for luciferase assays. As an internal control, the *Renilla* Luciferase construct pRL-TK (Promega) was used.

### Immunofluorescence Microscopy

Cells were fixed with 4% paraformaldehyde (PFA) for 20 min at 4°C, permeabilized with 0.05% Triton X-100 in PBS for 5 min at room temperature (RT), blocked with 5 mg/ml BSA in PBST (0.04% Tween20 in PBS) for 30 min, and incubated with relevant primary antibodies diluted in blocking buffer overnight at 4°C. The cells were then incubated with secondary antibodies for 1 h at RT. Nuclei were stained with 4.6-dimaidino-2-phenylinodole (DAPI) and analyzed with a confocal laser microscope, LSM 510-V4.2 (Carl Zeiss) or TCS-SP (Leica). The percentages of avSG-containing cells were calculated in more than 5 randomly chosen fields for each slide.

### Quantitative Reverse Transcription-PCR

Total RNA was prepared with TRIzol reagent (Invitrogen), treated with DNase I (Roche Applied Science), and amplified by reverse transcription-PCR with the ABI PRISM 7700 sequence detection system (Applied Biosystems). TaqMan reverse transcription reagents (Applied Biosystems) were used for cDNA synthesis. We used commercial TaqMan Universal PCR Master Mix and TaqMan primer-probe sets (Applied Biosystems) for human and mouse IFN-â. As an internal control for the comparative threshold cycle methods, a primer-probe set for eukaryotic 18 s rRNA (Applied Biosystems) was used. The results were normalized to the abundance of internal control. For the detection of IAV RNA and glyceraldehyde-3-phophate dehydrogenase (GAPDH), we used specific primer sets and amplified with Ex Taq HS (Takara). IAV RNA: 5¢- ATTTGCAACACTACAGGGGC-3¢ (forward) and 5¢-GACTGACGAAAGGAATCCCA-3¢ (reverse). GAPDH mRNA:

5¢- GAGTCAACGGATTTGGTCGT-3¢ (forward) and

5¢- TTGATTTTGGAGGGATCTCG-3¢ (reverse).

### Co-immunoprecipitation

The RIG-I antibody was cross-linked to Dynabeads protein G (Invitrogen) according to the manufacturer’s protocol. Cell lysate was incubated with the anti-RIG-I antibody -Dynabeads for 120 min at RT. RIG-I-immunoprecipitated complexes were eluted by boiling in loading buffer and then processed for Western blotting.

### RNA Interference

A lentiviral shRNA expression system was used. RIG-I shRNA#1 and RIG-I shRNA#2 were originally constructed. Oligonucleotides with the following sense and antisense sequences were used for the construction of the small hairpin RNA (shRNA)-encoding lentiviral vector. RIG-I shRNA#1; 5¢-GATCCCCGAGGTGCAGTATATTCAGGTT CAAGAGACCTGAATATACTGCACCTCTTTTTGGAAA-3¢ (sense) and 5¢-AGCTT T TCCAAAAAGAGGTGCAGTATATTCAGGTCTCTTGAACCTGAATATACTG CACCTCGGG-3¢ (antisense). RIG-I shRNA#2; 5¢-GATCCCCGAATTTAAAACCA GAATTATCTTCAAGAGAGATAATTCTGGTTTTAAATTCTTTTTGGAAA-3¢ (sense) and 5¢-AGCTTTTCCAAAAAGAATTTAAAACCAGAATTATCTCTC TTGAAGATAATTCTGGTTTTAAATTCGGG-3¢ (antisense). The oligonucleotides described above were annealed and subcloned into the Bgl II-Hind III site of pSUPER. To construct the pLV-shRNA against RIG-I, the BamHI-SalI fragments excised from pSUPER-RIG-I#1 and pSUPER-RIG-I#2 were subcloned into the BamHI-SalI site of pRDI292. The recombinant lentiviruses were generated by transfection of the empty lentiviral vector, or respective shRNA construct together with the packaging construct pCMVÄR8.91 and the envelop plasmid pMDG. At 48 h after, the culture supernatant was collected and the medium filtered with a 0.45-µm filter was transferred, to HeLa cells. After 72 h, the cells were selected with medium containing 2 µg/ml of Puromycin (Sigma). The siRNA negative control and siRNAs targeting PKR and G3BP were purchased from Invitrogen. Each siRNA was transfected with Lipofectamine RNAi MAX (Invitrogen) according to the manufacturer’s instructions. At 48 h post-transfection, cells were harvested, infected with IAVÄNS1, and then subjected to Real Time PCR, immunofluorescence assays, or SDS-PAGE followed by immunoblotting.

### Fluorescence in situ Hybridization (FISH**)** Assay

FISH assays have been described previously [57]. Briefly, after the immunofluorescence assays, cells were fixed in 4%PFA for 10 min and permeablized on ice with 0.5% Triton X-100 in PBS for 5 min. After deproteinization by Proteinase K, cells were re-fixed in 4% PFA for 10 min and then subjected to stepwise dehydration in ethanol. The dried coverslips were incubated with a biotin-labeled RNA probe for 12 h at 37°C. After hybridization, cells were washed and incubated with avidin-FITC for 30 min at 37°C. Nuclei were stained with TO-PRO-3 and examined by confocal laser-scanning microscope.

### Enzyme-linked Immunosorbent Assay (ELISA**)**

Culture supernatants were collected and subjected to ELISA with mouse IFN-â kit (PBL Interferon Source) according to the manufacturers’ instructions.

## Supporting Information

Figure S1 **Anti-RIG-I antibody specifically recognizes endogenous human RIG-I.** HeLa cells were infected with control lentivirus or two lentiviruses encoding different RIG-I-specific shRNAs (#1 and #2) for 72 h. **(A)** The cells were treated with NaAsO_2_ for 1 h and stained for RIG-I and G3BP. NaAsO_2_ induces speckle-like localization of RIG-I and G3BP. **(B)** The cells were treated with human IFN-â for 12 h. Cell extracts were prepared and subjected to SDS-PAGE, and immunoblotted using antibodies against RIG-I, MDA5, and â-actin. The RIG-I signals were diminished by knockdown of RIG-I.
(TIF)

Figure S2 **N-terminal region of NS1 is sufficient to block RIG-I aggregation and antiviral signals.** **(A)** 293T cells were transfected with empty vector (empty), myc-tagged NS1 (myc-NS1 (Full)), N-terminal NS1 (1–80), or C-terminal NS1 (81–230) for 48 h. The cells were mock-treated (mock) or infected with IAVÄNS1 for 9 h and stained for RIG-I and NS1 (myc). The percentage of cells with IAVÄNS1-induced RIG-I speckle was 0.0%, 2.3%, 43.1%, for NS1, NS1 (1–80), and NS1 (81–230)-expressing cells, respectively. **(B)** 293T cells were transiently transfected with reporter plasmids containing natural IFN-â promoter together with the indicated NS1-expressing vectors. Transfected cells were mock-treated or infected with IAVÄNS1 for 12 h and subjected to the Dual-Luciferase assay. Data are presented as the mean standard ± error of the mean (SEM).
(TIF)

Figure S3 **Localization of Viral RNA in IAV-infected cells. (A and B)** HeLa cells were mock-treated or infected with IAV for 12 h. Viral RNA (vRNA) was detected by the FISH method using an RNA probe complementary to the segment 1 of IAV and NP **(A)** and RIG-I **(B)** were detected using anti-NP and anti-RIG-I antibodies. TO-PRO-3 was used for staining of nuclear DNA (DNA). Viral RNA and NP did not form foci.
(TIF)

Figure S4 **IPS-1 was accumulated in close proximity to the RIG-I foci.** HeLa cells stably expressing FLAG-tagged IPS-1 were mock-treated or infected with IAV or IAVÄNS1 for 10 h. The cells were stained with anti-FLAG and anti-RIG-I antibodies and DAPI. The merged images of FLAG and RIG-I are enlarged in the bottom panel. The white arrowheads indicate RIG-I/IPS-1 contacts. These contacts were observed in 74.2% and 1.8% of IAVÄNS1- and IAV-infected cells, respectively.
(TIF)

Figure S5 **avSG formation is not a consequence of IFN gene activation.** **(A)** 293T cells were transfected with empty vector or the expression vector for IPS-1 (HA-IPS-1) for 24 h. Cells were stained for IRF-3, HA-tag and TIAR. Nuclear IRF-3 was observed in almost all of the IPS-1-expressing cells (95.5%), however these cells exhibited little foci of TIAR (3.0%). **(B)** HEC-1B cells deficient for type I IFN receptor were mock-treated, infected with IAVÄNS1 for 9 h, or treated with NaAsO_2_ for 1 h as indicated. Cells were stained for RIG-I and G3BP. SGs and avSGs were observed in HEC-1B cells (% colocalization 98.4% and 92.9% for IAVÄNS1 and NaAsO_2_, respectively). The zoomed images correspond to the boxed regions. **(C)** HeLa cells were mock-treated or infected with SINV, EMCV, or Ad12 dl203 for 9 h, fixed, and stained for RIG-I and G3BP as indicated (% colocalization: 99.2%, 98.4%, and 98.2%, respectively). The zoomed images correspond to the boxed regions.
(TIF)

Figure S6 **IAV-induced formation of avSGs was inhibited in PKR knockdown cells.** **(A–E)** HeLa cells were transfected with control siRNA (N.C) or siRNA targeting three independent parts of human PKR mRNA (#1 3). **(A)** At 48 h after transfection, cells were harvested and PKR and actin were detected by Western blotting. **(B–E)** At 48 h after transfection, cells were mock-treated (open bar) or infected with IAVÄNS1 (filled bar) for 9 h. The level of IFN-â mRNA was determined by qPCR **(B)**. Immunostaining of HeLa cells transfected with control (N.C) or PKR-targeted (PKR) siRNA after mock-treatment or infection with IAVÄNS1 **(C)**. Cells were also examined by staining for foci of RIG-I **(D)**, TIAR **(E)** after 12 h infection. Percentages of cells containing the respective foci are indicated. Data are presented as the mean standard ± error of the mean (SEM).
(TIF)

Figure S7 **Total RNA from IAV-infected cells but not uninfected cells induces avSG formation and IFN-â gene activation. (A and B)** Wild-type MEF were mock-treated (no RNA) or transfected with total RNA extracted from uninfected MEFs (MEF RNA) or from IAV-infected cells for 12 h (IAV infected MEF RNA). The cells were stained for RIG-I and G3BP (% avSG formation 0.0%, 4.0%, and 21.4% for no RNA, MEF RNA, and IAV infected MEF RNA, respectively) **(A)**. The zoomed images correspond to the boxed regions. Endogenous IFN-â mRNA levels were determined by qPCR **(B)**. Data are presented as the mean standard ± error of the mean (SEM).
(TIF)
