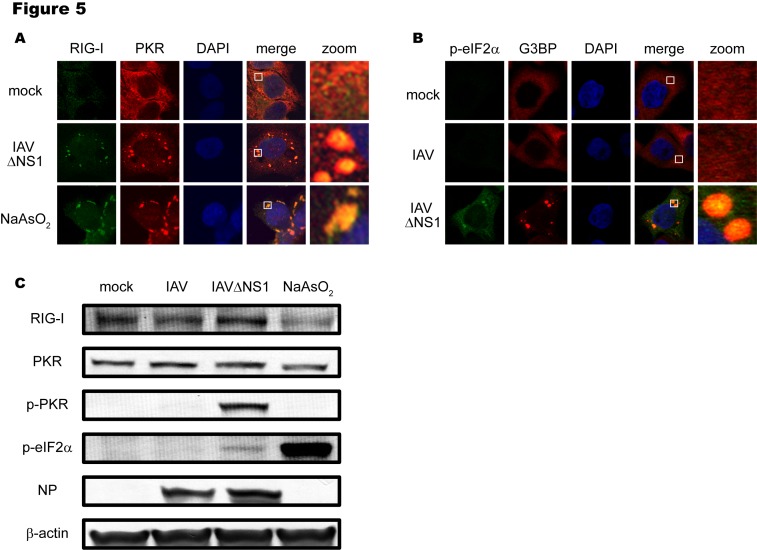# Correction: Critical Role of an Antiviral Stress Granule Containing RIG-I and PKR in Viral Detection and Innate Immunity

**DOI:** 10.1371/annotation/dcd836ee-9e23-4538-acb7-450560ba5c1d

**Published:** 2012-10-31

**Authors:** Koji Onomoto, Michihiko Jogi, Ji-Seung Yoo, Ryo Narita, Shiho Morimoto, Azumi Takemura, Suryaprakash Sambhara, Atushi Kawaguchi, Suguru Osari, Kyosuke Nagata, Tomoh Matsumiya, Hideo Namiki, Mitsutoshi Yoneyama, Takashi Fujita

There is an error in Figure 5. The correct figure can be viewed here: 

**Figure:**