# Correction: Critical Role of an Antiviral Stress Granule Containing RIG-I and PKR in Viral Detection and Innate Immunity

**DOI:** 10.1371/annotation/14a6b875-ed9d-41ae-8ce6-52e8c5c7f85b

**Published:** 2013-04-18

**Authors:** Koji Onomoto, Michihiko Jogi, Ji-Seung Yoo, Ryo Narita, Shiho Morimoto, Azumi Takemura, Suryaprakash Sambhara, Atushi Kawaguchi, Suguru Osari, Kyosuke Nagata, Tomoh Matsumiya, Hideo Namiki, Mitsutoshi Yoneyama, Takashi Fujita

Throughout the article multiple symbols were erroneous presented in incorrect forms - the correct versions are as follows:

á should be α

ê should be κ

Ä should be Δ

â should be β

¢ should be ‘ 

