# Supplementary figures and images for: Critical Role of an Antiviral Stress Granule Containing RIG-I and PKR in Viral Detection and Innate Immunity
Source: PLoS One. 2012 Aug 13;7(8):e43031. doi: 10.1371/journal.pone.0043031 (PMC3418241; doi:10.1371/journal.pone.0043031)

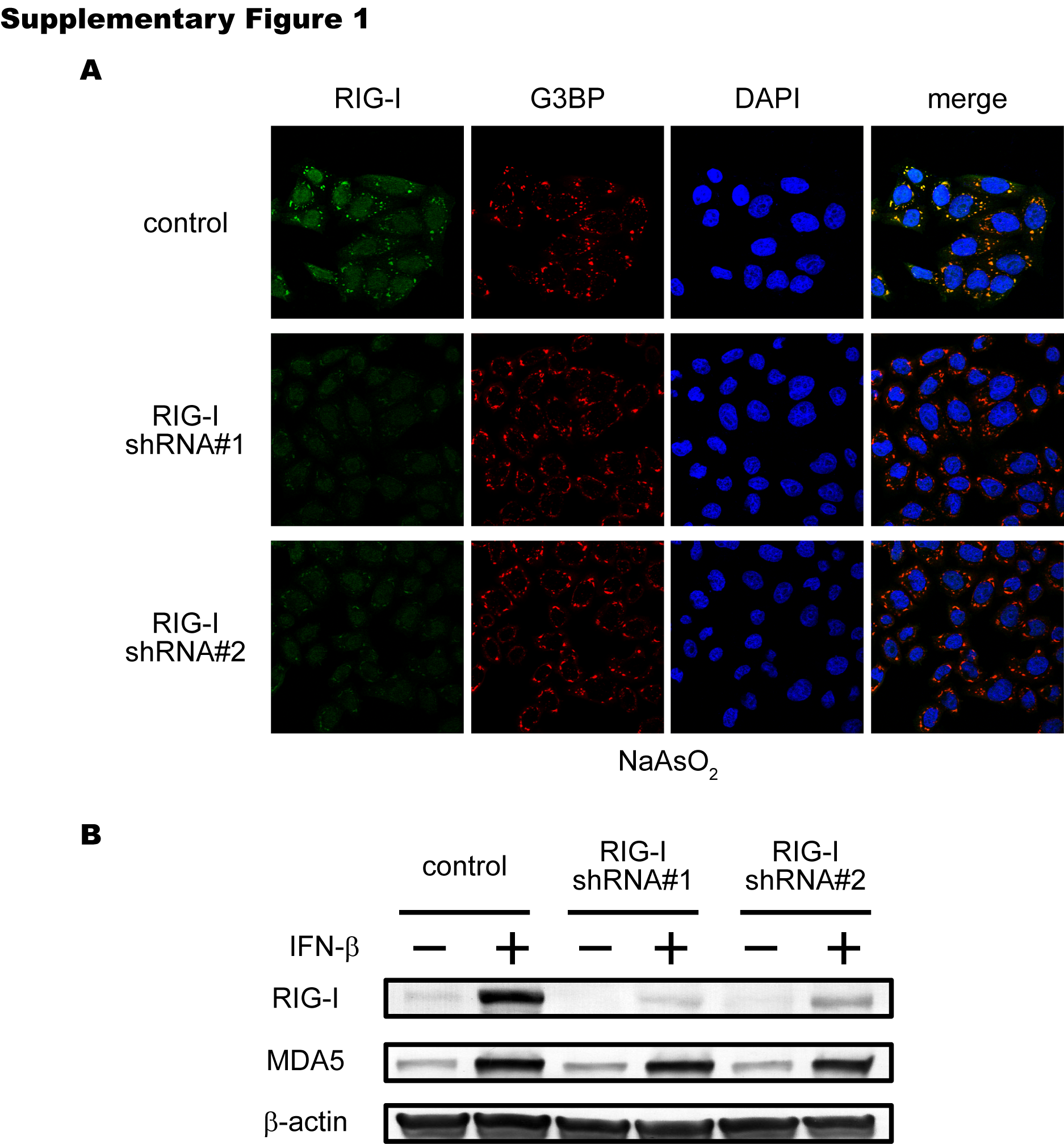

Supplement: Figure S1 — Anti-RIG-I antibody specifically recognizes endogenous human RIG-I. HeLa cells were infected with control lentivirus or two lentiviruses encoding different RIG-I-specific shRNAs (#1 and #2) for 72 h. (A) The cells were treated with NaAsO2 for 1 h and stained for RIG-I and G3BP. NaAsO2 induces speckle-like localization of RIG-I and G3BP. (B) The cells were treated with human IFN-â for 12 h. Cell extracts were prepared and subjected to SDS-PAGE, and immunoblotted using antibodies against RIG-I, MDA5, and â-actin. The RIG-I signals were diminished by knockdown of RIG-I. (TIF) [file pone.0043031.s001.tif]

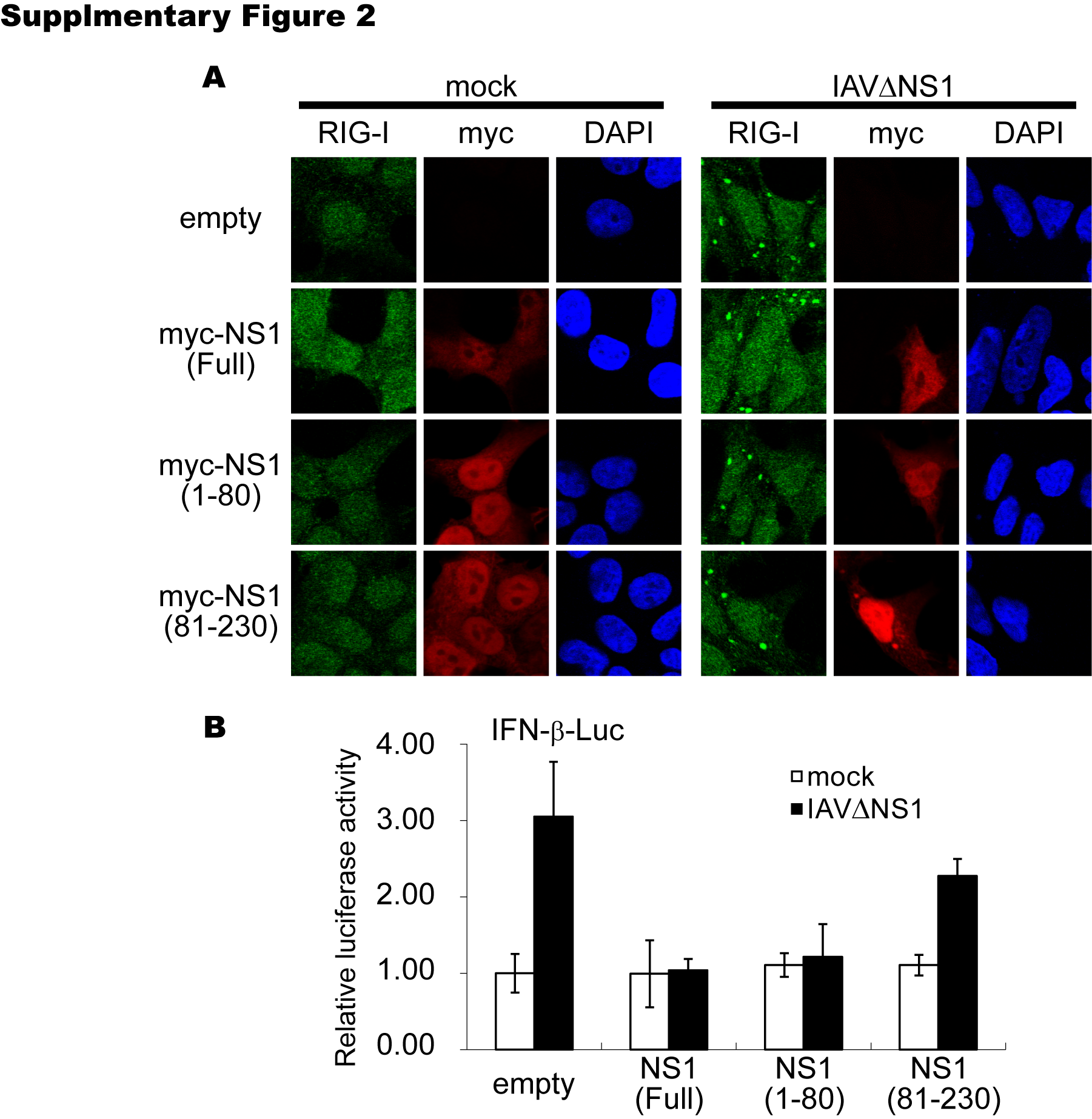

Supplement: Figure S2 — N-terminal region of NS1 is sufficient to block RIG-I aggregation and antiviral signals. (A) 293T cells were transfected with empty vector (empty), myc-tagged NS1 (myc-NS1 (Full)), N-terminal NS1 (1–80), or C-terminal NS1 (81–230) for 48 h. The cells were mock-treated (mock) or infected with IAVÄNS1 for 9 h and stained for RIG-I and NS1 (myc). The percentage of cells with IAVÄNS1-induced RIG-I speckle was 0.0%, 2.3%, 43.1%, for NS1, NS1 (1–80), and NS1 (81–230)-expressing cells, respectively. (B) 293T cells were transiently transfected with reporter plasmids containing natural IFN-â promoter together with the indicated NS1-expressing vectors. Transfected cells were mock-treated or infected with IAVÄNS1 for 12 h and subjected to the Dual-Luciferase assay. Data are presented as the mean standard ± error of the mean (SEM). (TIF) [file pone.0043031.s002.tif]

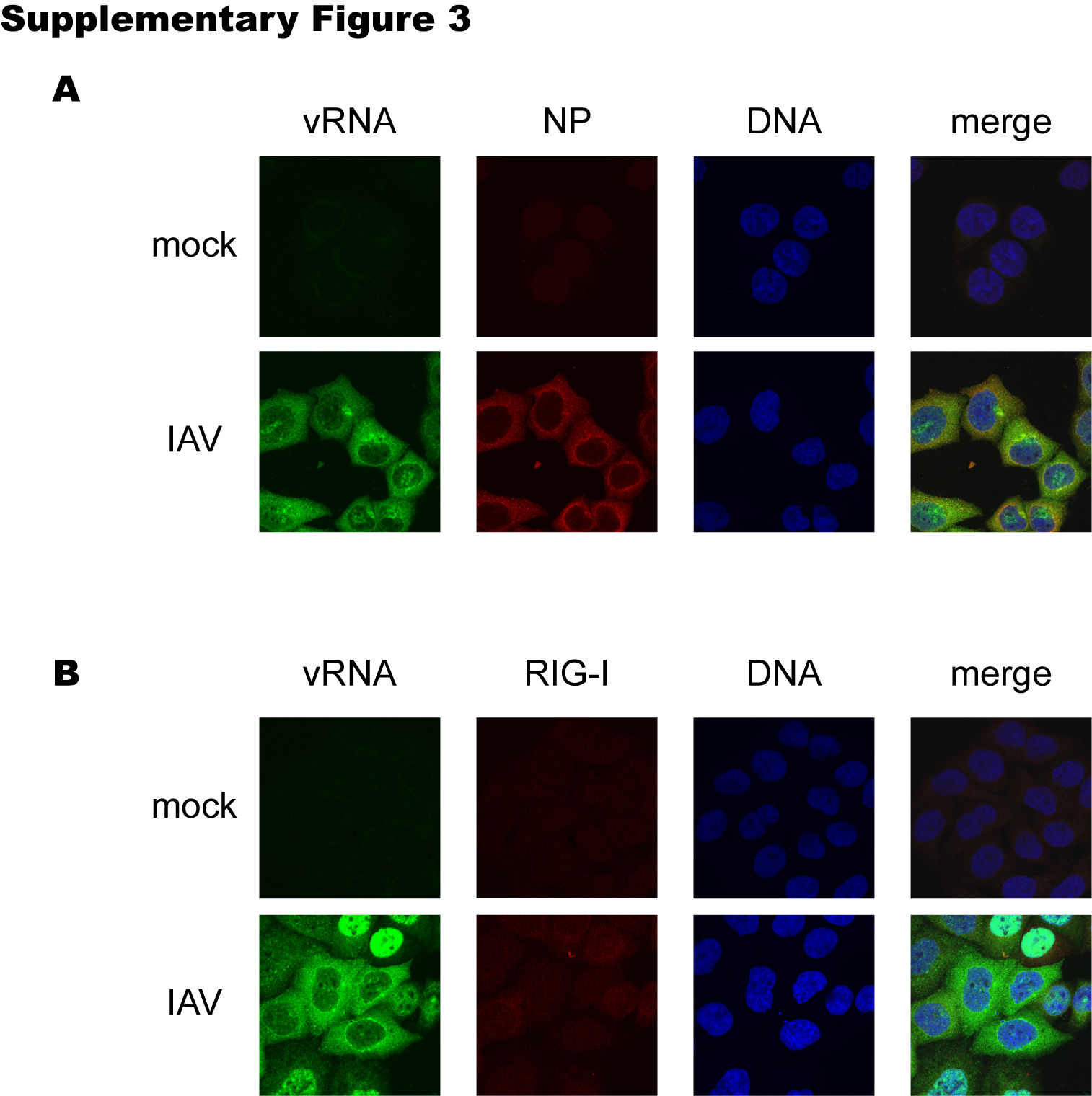

Supplement: Figure S3 — Localization of Viral RNA in IAV-infected cells. (A and B) HeLa cells were mock-treated or infected with IAV for 12 h. Viral RNA (vRNA) was detected by the FISH method using an RNA probe complementary to the segment 1 of IAV and NP (A) and RIG-I (B) were detected using anti-NP and anti-RIG-I antibodies. TO-PRO-3 was used for staining of nuclear DNA (DNA). Viral RNA and NP did not form foci. (TIF) [file pone.0043031.s003.tif]

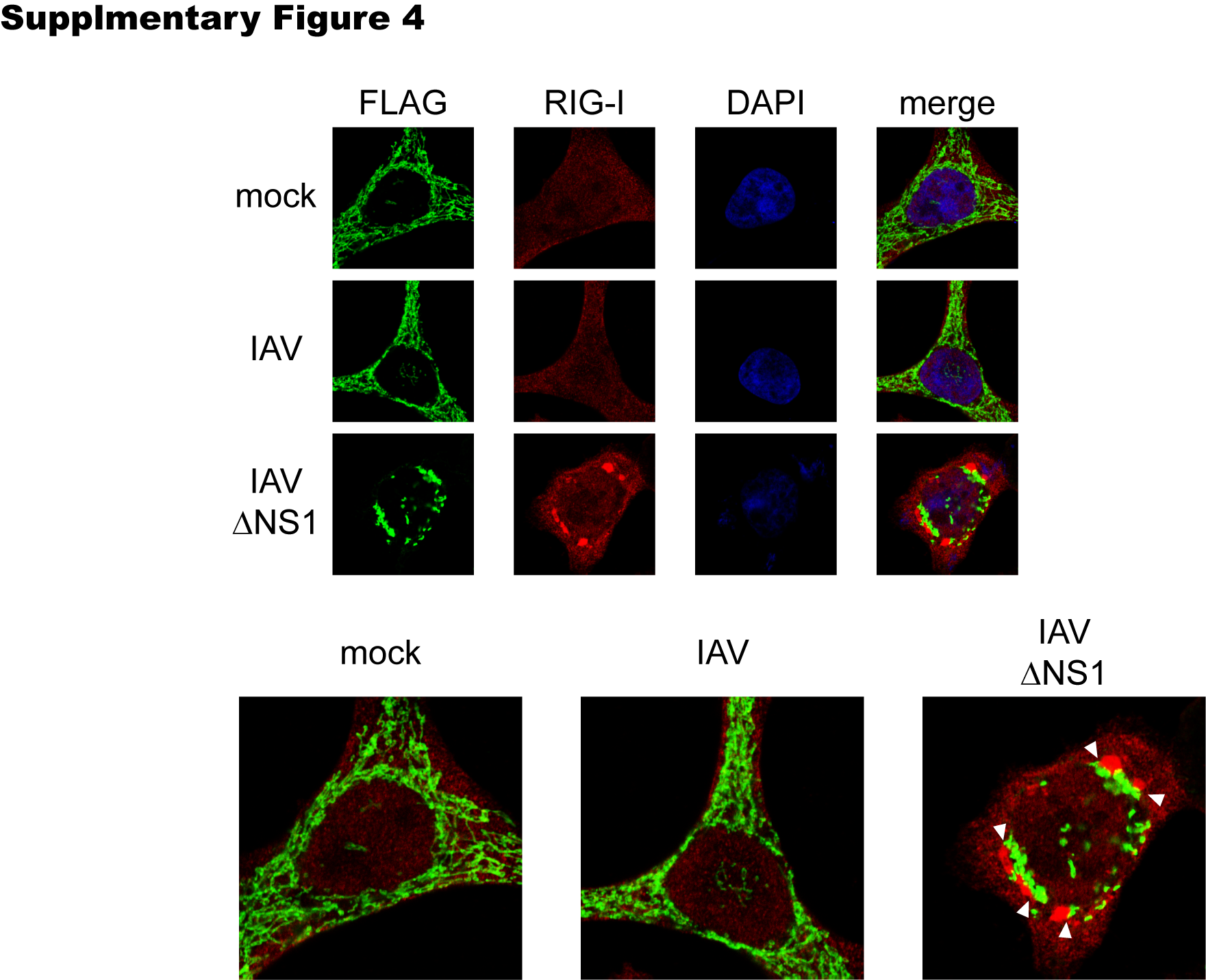

Supplement: Figure S4 — IPS-1 was accumulated in close proximity to the RIG-I foci. HeLa cells stably expressing FLAG-tagged IPS-1 were mock-treated or infected with IAV or IAVÄNS1 for 10 h. The cells were stained with anti-FLAG and anti-RIG-I antibodies and DAPI. The merged images of FLAG and RIG-I are enlarged in the bottom panel. The white arrowheads indicate RIG-I/IPS-1 contacts. These contacts were observed in 74.2% and 1.8% of IAVÄNS1- and IAV-infected cells, respectively. (TIF) [file pone.0043031.s004.tif]

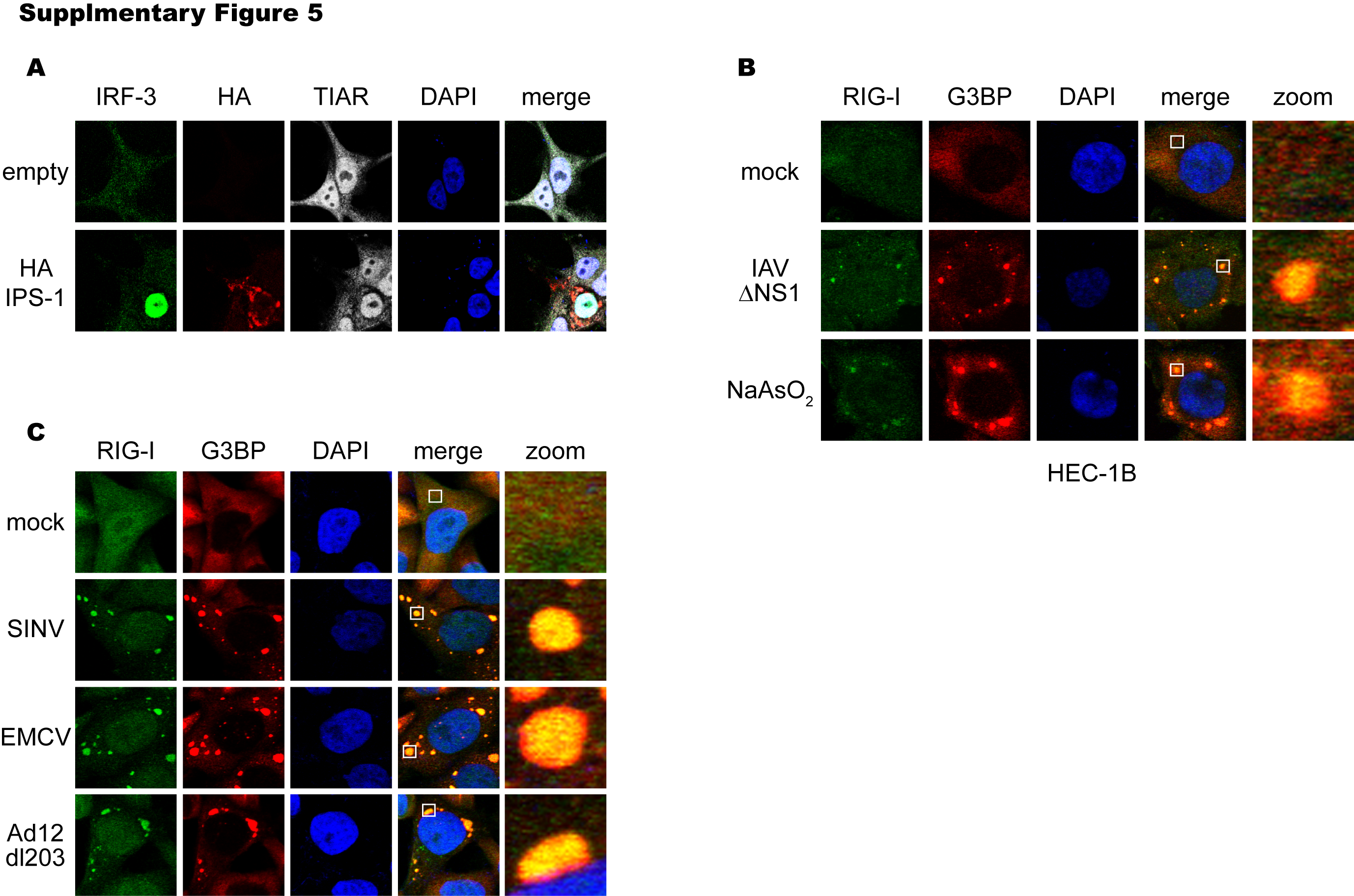

Supplement: Figure S5 — avSG formation is not a consequence of IFN gene activation. (A) 293T cells were transfected with empty vector or the expression vector for IPS-1 (HA-IPS-1) for 24 h. Cells were stained for IRF-3, HA-tag and TIAR. Nuclear IRF-3 was observed in almost all of the IPS-1-expressing cells (95.5%), however these cells exhibited little foci of TIAR (3.0%). (B) HEC-1B cells deficient for type I IFN receptor were mock-treated, infected with IAVÄNS1 for 9 h, or treated with NaAsO2 for 1 h as indicated. Cells were stained for RIG-I and G3BP. SGs and avSGs were observed in HEC-1B cells (% colocalization 98.4% and 92.9% for IAVÄNS1 and NaAsO2, respectively). The zoomed images correspond to the boxed regions. (C) HeLa cells were mock-treated or infected with SINV, EMCV, or Ad12 dl203 for 9 h, fixed, and stained for RIG-I and G3BP as indicated (% colocalization: 99.2%, 98.4%, and 98.2%, respectively). The zoomed images correspond to the boxed regions. (TIF) [file pone.0043031.s005.tif]

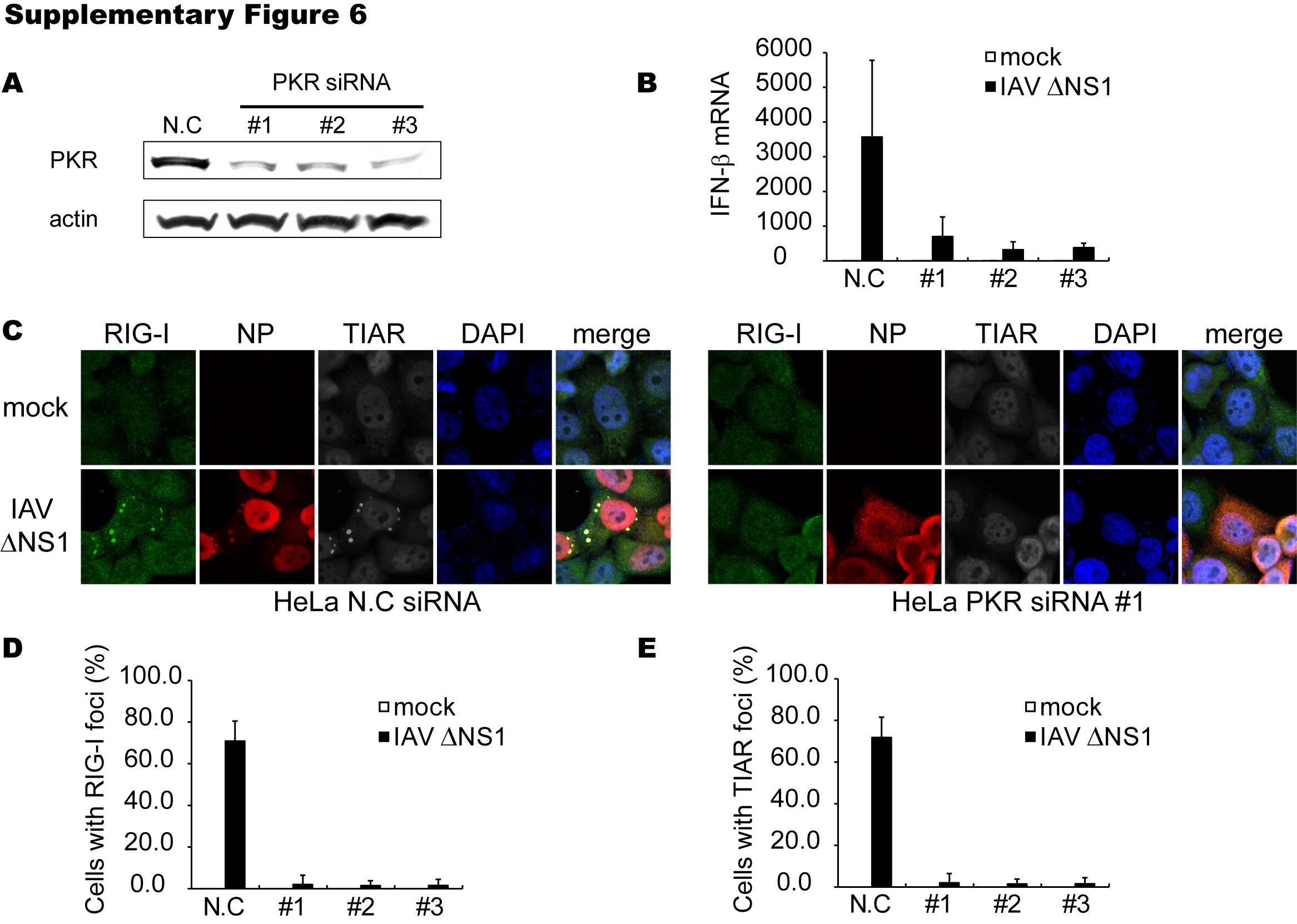

Supplement: Figure S6 — IAV-induced formation of avSGs was inhibited in PKR knockdown cells. (A–E) HeLa cells were transfected with control siRNA (N.C) or siRNA targeting three independent parts of human PKR mRNA (#1 3). (A) At 48 h after transfection, cells were harvested and PKR and actin were detected by Western blotting. (B–E) At 48 h after transfection, cells were mock-treated (open bar) or infected with IAVÄNS1 (filled bar) for 9 h. The level of IFN-â mRNA was determined by qPCR (B). Immunostaining of HeLa cells transfected with control (N.C) or PKR-targeted (PKR) siRNA after mock-treatment or infection with IAVÄNS1 (C). Cells were also examined by staining for foci of RIG-I (D), TIAR (E) after 12 h infection. Percentages of cells containing the respective foci are indicated. Data are presented as the mean standard ± error of the mean (SEM). (TIF) [file pone.0043031.s006.tif]

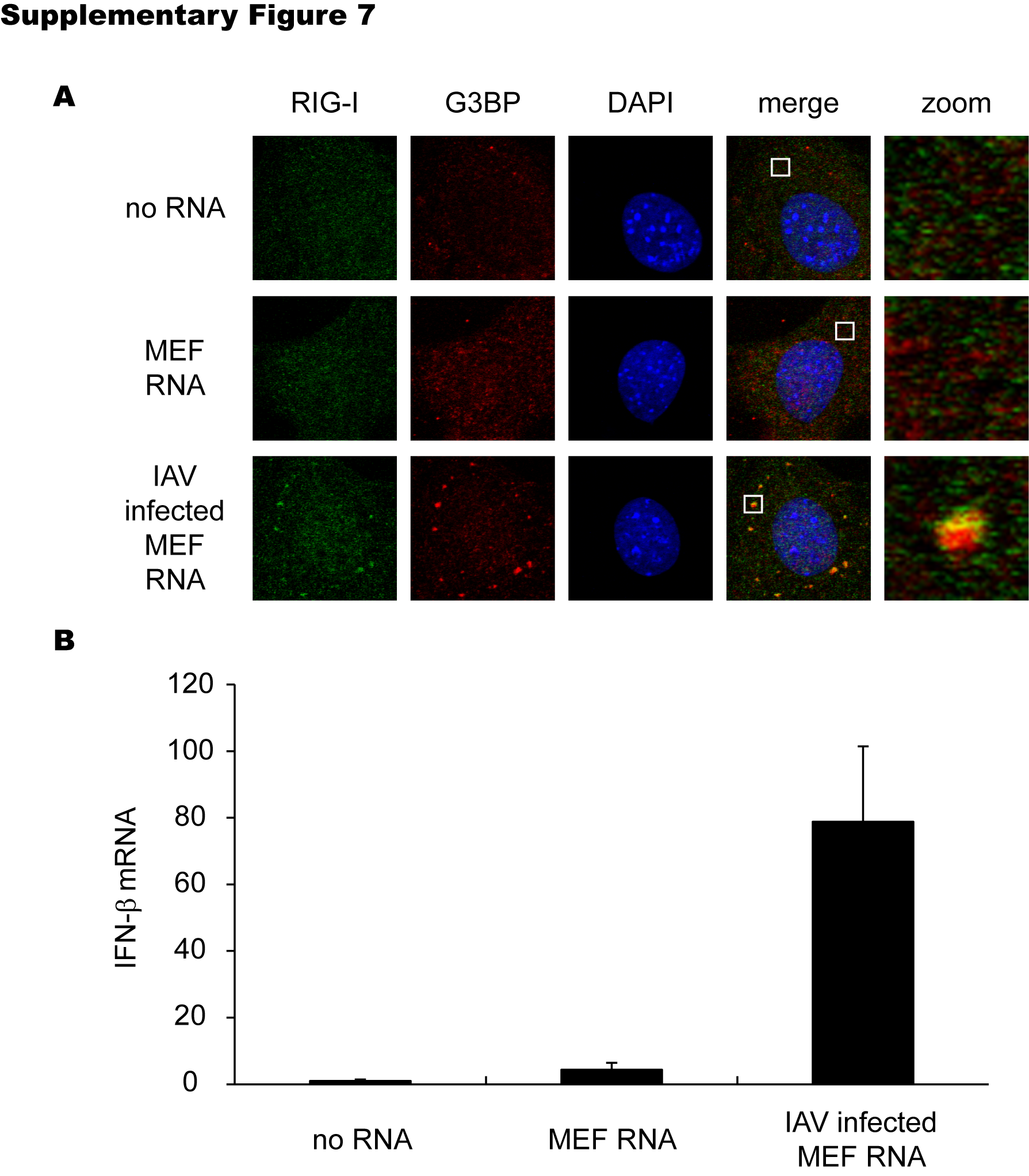

Supplement: Figure S7 — Total RNA from IAV-infected cells but not uninfected cells induces avSG formation and IFN-â gene activation. (A and B) Wild-type MEF were mock-treated (no RNA) or transfected with total RNA extracted from uninfected MEFs (MEF RNA) or from IAV-infected cells for 12 h (IAV infected MEF RNA). The cells were stained for RIG-I and G3BP (% avSG formation 0.0%, 4.0%, and 21.4% for no RNA, MEF RNA, and IAV infected MEF RNA, respectively) (A). The zoomed images correspond to the boxed regions. Endogenous IFN-â mRNA levels were determined by qPCR (B). Data are presented as the mean standard ± error of the mean (SEM). (TIF) [file pone.0043031.s007.tif]
